# Chloroform Extract of Rasagenthi Mezhugu, a Siddha Formulation, as an Evidence-Based Complementary and Alternative Medicine for HPV-Positive Cervical Cancers

**DOI:** 10.1155/2012/136527

**Published:** 2011-10-27

**Authors:** Anvarbatcha Riyasdeen, Vaiyapuri S. Periasamy, Preethy Paul, Ali A. Alshatwi, Mohammad A. Akbarsha

**Affiliations:** ^1^Department of Animal Science, Bharathidasan University, Tiruchirappalli 620 024, India; ^2^Department of Food Science and Nutrition, College of Food and Agricultural Sciences, King Saud University, Riyadh 11451, Saudi Arabia; ^3^Mahatma Gandhi-Doerenkamp Center for Alternatives to Use of Animals in Life Science Education, Bharathidasan University, Tiruchirappalli 620 024, India

## Abstract

Rasagenthi Mezhugu (RGM) is a herbomineral formulation in the Siddha system of traditional medicine and is prescribed in the southern parts of India as a remedy for all kinds of cancers. However, scientific evidence for its therapeutic efficacy in cervical cancer is lacking, and it contains heavy metals. To overcome these limitations, RGM was extracted, and the fractions were tested on HPV-positive cervical cancer cells, ME-180 and SiHa. The extracts, free from the toxic heavy metals, affected the viability of both the cells. The chloroform fraction (cRGM) induced DNA damage and apoptosis. Mitochondria-mediated apoptosis was indicated. Though both the cells responded to the treatment, ME-180 was more responsive. Thus, this study brings up scientific evidence for the efficacy of RGM against the HPV-mediated cervical cancer cells and, if the toxic heavy metals are the limitation in its use, cRGM would be a suitable candidate as evidence-based complementary and alternative medicine for HPV-positive cervical cancers.

## 1. Introduction

 Cancer is one of the major public health problems worldwide and accounts for an estimated 2.5 million cases in India alone [1]. In the wake of resistance to chemotherapy and the escalating toxic effects of synthetic drugs/compounds, all possible avenues are being explored to develop new and novel anticancer drugs that will overcome these limitations. One of the avenues is phytotherapy, which is a recognized complementary and alternative (CAM) therapeutic modality [2]. Many cancer patients, who are already crippled with this disease, and further burdened by drug-induced toxic side effects, now turn to complementary and alternative medicines hoping for a better cure or at least palliation [3]. Herbalism is a common medical practice since time immemorial. More than 60% of the approved drugs are derived from nature, and most of these discoveries were led from traditional herbal medicines [4]. The Indian traditional systems of medicine and folk medicines make use of thousands of plant-based formulations [5]. The principle underlying the use of more than one plant/plant product in these formulations is that they may produce synergistic and/or additive effects, or one may neutralize the toxic effect of another, which is otherwise therapeutic in the given context [6]. 

 Siddha is one among the three popular Indian traditional medicinal systems, the other two being Ayurveda and Unani. Siddha medicine formulations are mostly polyherbal, but may also include metals, chemicals, and/or animal products. The common Siddha preparations are Bhasma (calcined metals and minerals), Churna (powders), Kashaya (decoctions), Lehya (confections), Ghrita (ghee), Taila (oil), and Mezhugu (wax). Rasagenthi Mezhugu (RGM), a Siddha medicine, is a formulation containing 38 different botanicals and 8 inorganic substances, some of which are heavy metals [7]. Siddha practitioners prescribe RGM as a therapy for different cancers [7]. However, scientific evidence for the therapeutic efficacy of RGM in cancer is far too limited. This is in view of the fact that the complexity of the formulation does not facilitate investigations *in vitro*. Also, the heavy metals in RGM, mercury, lead, and arsenic, are toxic [8]. To overcome both these limitations, a modality was developed whereupon RGM was extracted in solvents of increasing polarity, and the extracts were tested on cancer cell lines [7, 9]. The extracts were found to be free from the toxic heavy metals and amenable for *in vitro* testing. Thus, chloroform fraction of RGM was shown to be cytotoxic to prostate cancer cell PC3 [7] and lung cancer cells A-549 and H-460 [9], and in both cases, the cells succumbed to death by apoptosis.

 Cervical cancer is one of the serious health problems in women [10]. In India alone, more than 70,000 new cases of cervical cancer are reported every year [1]. Most of the cervical cancers are caused by HPV infection and integration of HPV genome into the host cell's genome [11]. Thus, these cervical cancers being etiologically different, it would be pertinent to find if prescription of RGM to cervical cancer patients can have a scientific backing. Therefore, we carried out this study to test the most efficacious extract of RGM, the chloroform extract, which is free from heavy metals [7, 9], on HPV-mediated cervical cancer cell lines, ME-180 and SiHa. In doing so, we focused on apoptosis as the end point, since in these cervical cancer cells, the cell cycle progression and apoptosis cascade are deregulated [12]. 

## 2. Materials and Methods

### 2.1. Preparation of RGM

Rasagenthi Mezhugu was obtained from Indian Medical Practitioners Co-operative Pharmacy and Stores Ltd., (IMPCOPS, Thiruvanmiyur, Chennai, India), an authoritative source of Indian medicines (http://www.impcops.org/), and its composition has been already described [7].

### 2.2. Extraction of RGM

 The extraction procedure also has been previously described [7]. Briefly, RGM was extracted with methanol (MeOH) using Soxhlet apparatus. The MeOH phase was evaporated under reduced pressure to obtain a dark brown residue. This residue was suspended in water and extracted with four organic solvents with increasing polarity, namely, *n*-hexane, CHCl_3_, EtOAc, and *n*-BuOH, and the final residue was extracted in water. All five extracts were condensed into powder/paste under reduced pressure using a rotary evaporator (Buchi Labortechnik AG, Flawil, Switzerland).

### 2.3. Cell Culture

Human cervical cancer cells ME-180 and SiHa were obtained from National Center for Cell Science (NCCS), Pune, India. The cells were maintained in DMEM medium supplemented with 10% FBS (Sigma-Aldrich, St. Louis, Mo, USA), and with 100 U/mL penicillin and 100 *μ*g/mL streptomycin as antibiotics (Himedia, Mumbai, India) in a humidified atmosphere of 5% CO_2_ and 95% air in a CO_2_ incubator (Heraeus, Hanau, Germany).

### 2.4. Cell Viability Assay

All five RGM extracts, in the concentration range of 0–500 *μ*g/mL, dissolved in DMSO (Sigma-Aldrich), were added to the wells, 24 h after seeding of 5 × 10^3^ cells per well of 96-well plate. DMSO was used as the solvent control. After 24 and 48 h of incubation, 20 *μ*L of MTT solution (5 mg/mL in phosphate-buffered saline (PBS)) was added to each well, and the plates were wrapped with aluminum foil and incubated for 4 h at 37°C. The purple formazan product was dissolved by addition of 100 *μ*L of 100% DMSO to each well. The absorbance was monitored at 570 nm (measurement) and 630 nm (reference) using a 96-well plate reader (Bio-Rad, Hercules, Calif, USA). Data were collected for four replicates each and used to calculate the means and the standard deviations. The percentage inhibition was calculated from this data using the following formula:

(1) $$\text{The}\;\text{percentage}\;\text{inhibition}=\frac{\text{Mean}\;\text{OD}\;\text{of}\;\text{untreated}\;\text{cells}\;\left(\text{control}\right)-\text{Mean}\;\text{OD}\;\text{of}\;\text{treated}\;\text{cells}}{\text{Mean}\;\text{OD}\;\text{of}\;\text{untreated}\;\text{cells}\;\left(\text{control}\right)}\;\times 100.$$

From the values thus obtained, the IC_50_ for the respective extracts, and for the respective durations of treatment, that is, 24 and 48 h, was deduced from the curves obtained by plotting percentage inhibition against concentration. Since the MTT test indicated that the chloroform extract of RGM was the most efficacious among the five extracts, and it affected the viability of the cells at concentrations very low compared to the others, subsequent studies were limited to this extract (cRGM).

### 2.5. Hoechst 33528 Staining

 The cervical cancer cells ME-180 and SiHa were cultured in 6-well plates and treated with IC_50_ concentration of cRGM. After 24 and 48 h incubation, the treated and untreated cells were harvested and stained with Hoechst 33258 (1 mg/mL, aqueous) for 5 min at room temperature. A drop of cell suspension was placed on a glass slide, and a cover slip was laid over to reduce light diffraction. At random 300 cells, in duplicate, were observed at ×400 in a fluorescent microscope (Carl Zeiss, Jena, Germany) fitted with a 377–355 nm filter, and the percentage of cells reflecting pathological changes was calculated.

### 2.6. Acridine Orange (AO) and Ethidium Bromide (EB) Fluorescent Assay for Cell Death

Acridine orange (AO) and ethidium bromide (EB) staining was performed as described by Spector et al. [13]. The cells were cultured in 6-well plates and treated with IC_50_ concentration of cRGM for 24 and 48 h. The treated and untreated cells (25 *μ*L of suspension containing 5  ×  10^5^ cells) were incubated with acridine orange and ethidium bromide solution (1 part of 100 *μ*g/mL acridine orange and 1 part of 100 *μ*g/mL ethidium bromide in PBS) and examined in the fluorescent microscope using a UV filter (450–490 nm). Three hundred cells per sample were counted, in duplicate, for each time point (24, 48 h). The cells were scored as viable or dead, and if dead, whether by apoptosis or necrosis as judged from nuclear morphology and cytoplasmic organization. The percentages of apoptotic and necrotic cells were then calculated. Morphological features of interest were photographed.

### 2.7. Single-Cell Gel Electrophoresis (Comet Assay)

 DNA damage was detected by adopting the comet assay [14]. Treated (IC_50_ concentration; 24 and 48 h treatment) and control cells were suspended in low-melting-point agarose in PBS and pipetted on to microscope slides precoated with a layer of normal-melting-point agarose. The slides were chilled on ice for 10 min and then immersed in lysis solution (2.5 M NaCl, 100 × 10^−3^ M Na_2_EDTA, 10 × 10^−3^ M Tris, 0.2 × 10^−3^ M NaOH, pH 10.01, and Triton X-100), and the solution was kept overnight at 4°C in order to lyse the cells and to permit DNA unfolding. The slides were then exposed to alkaline buffer (300 × 10^−3^ M NaOH, 1 × 10^−3^ M Na_2_EDTA, pH > 13) for 20 min to allow DNA unwinding. The slides were washed with buffer (0.4 M Tris, pH 7.5) to neutralize excess alkali and to remove detergents, before staining with EB. Photomicrographs were obtained using the fluorescent microscope. One hundred cells, in duplicate, from each treatment group were digitalized and analyzed using Comet Assay Software Program (*CASP*). The images were used to estimate the DNA content of individual nuclei and to evaluate the degree of DNA damage that represented the fraction of total DNA in the tail.

### 2.8. Assay for Mitochondrial Transmembrane Potential

Mitochondrial transmembrane potential was assessed using the fluorescent probe JC-1, which produces green fluorescence in the cytoplasm and red-orange fluorescence when accumulated in healthy mitochondria. In case the mitochondrial membrane potential is affected, JC1 will be limited to cytoplasm, and the whole cell will fluoresce green. The cells were grown in six well plates and treated with IC_50_ concentration of cRGM. After 12 and 24 h exposure, the cells were stained for 30 min with JC-1 (2 *μ*g/mL) in the culture medium. The adherent cell layer was then washed with PBS and lifted using 250 *μ*L of trypsinEDTA. The cells were collected in PBS, washed by centrifugation, resuspended in 0.3 mL of PBS, mixed gently, and examined in the fluorescent microscope using a UV filter (450–490 nM). The specific fluorescent patterns were indicative of intact (red fluorescence) or loss (green fluorescence) of mitochondrial transmembrane potential (ΔΨm). 

### 2.9. Annexin V-Cy3 Apoptosis Assay

Phosphatidylserine translocation from inner to outer leaflet of the plasma membrane is one of the early features of apoptosis. Cell surface phosphatidylserine was detected using phosphatidylserine-binding protein annexin V conjugated with Cy3 using the commercially available annexin V-Cy3 apoptosis detection kit (APOAC, Apoptosis Detection Kit, Sigma, Calif, USA). The cells were treated with IC_50_ concentration of cRGM. After 12 and 24 h incubation, the cells were harvested, centrifuged, and pellets were collected. The cell pellet was washed with PBS and then with 1x binding buffer. The washed cell pellet was suspended in 50 *μ*L of double-label staining solution (Ann-Cy3 and 6-CFDA) and kept in dark for 10 min. After the incubation, the excess label was removed by washing the cells with 1x binding buffer. The annexin-Cy3 and 6-CFDA-labelled cells were observed in the fluorescent microscope. 300 cells at random were observed. This assay facilitated detection of live cells (green), necrotic cells (red), and apoptotic cells (red nuclei and green cytoplasm). The percentage of cells reflecting cell death (apoptotic and necrotic, separately) was calculated. Data were collected from two individual experiments, each in duplicate, and used to calculate the respective means and the standard deviations.

### 2.10. Statistics

Numerical data are expressed as mean ± standard deviation (SD). Statistical differences were evaluated by a one-way analysis of variance (ANOVA) using statistical package for social sciences (SPSS) software for window9 Version 11.5 (SPSS) Inc., Chicago, Ill, USA). Posthoc test was performed for comparisons using the least significant difference (LSD) test. Differences were considered statistically significant when *P* < 0.05.

## 3. Results

### 3.1. Effect of cRGM on Viability of Cells as Revealed in MTT Assay

MTT assay determines the integrity of mitochondria and reflects the viability or otherwise of the cells. The results of MTT assay showed that although all extracts of cRGM, other than water extract, inhibited proliferation of both SiHa and ME-180 cervical cancer cells in time- and dose-dependent manner, cRGM was the most efficacious since it affected viability of the cells at a concentration many times lesser than the others (Table 1). Of the two cell types subjected to the test, ME-180 was more responsive than SiHa. Therefore, the rest of the study was limited to cRGM.

### 3.2. Changes in Nucleus and Chromatin as Revealed in Hoechst Staining

**Figure 1:**
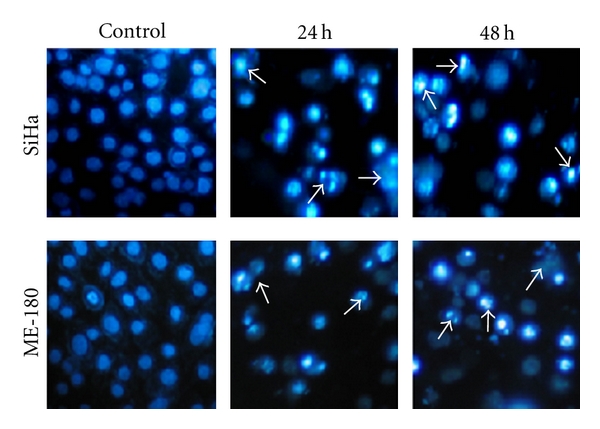
Control and cRGM-treated (24 h, 48 h) SiHa and ME-180 cells stained with Hoechst 33258, and arrow marks indicate apoptotic cells.

Hoechst 33528 staining showed that there were significant changes in the chromatin of treated cells. In the untreated cells, the nuclei were round, even, and homogenous, and the chromatin was intact. After treatment with cRGM for 24 and 48 h, the intensity of blue fluorescence emittance in respect of the treated cells was much brighter than the control cells, and changes in the chromatin such as condensation, marginalization, and fragmentation were observed (Figure 1). 

**Figure 2:**
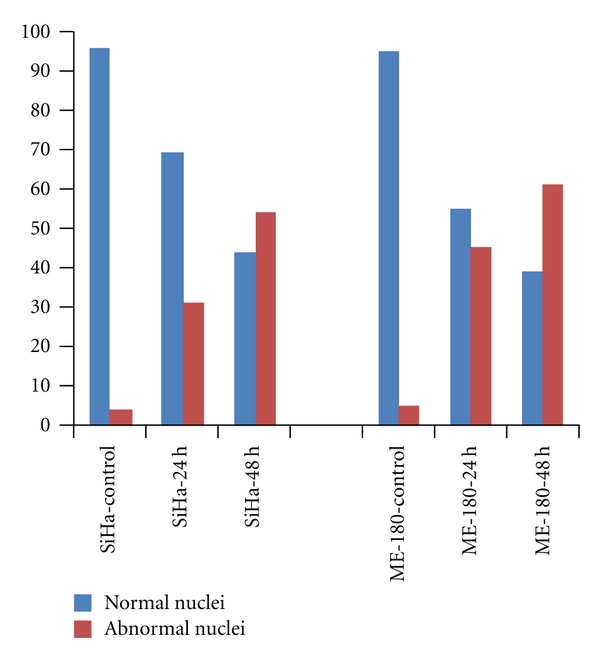
Percentage of cells with normal and abnormal nuclei at 24 and 48 h in control and cRGM-treated cells.

 The nuclei were found to be abnormal in 31% and 54% of cRGM-treated SiHa cells in the 24, 48 h treatment groups, respectively. In the case of ME-180 cell, the impact was much higher, since 45% and 61% of cells were affected during 24 and 48 h treatment, respectively (Figure 2)

### 3.3. Apoptotic versus Necrotic Death Caused by the Treatment

**Figure 3:**
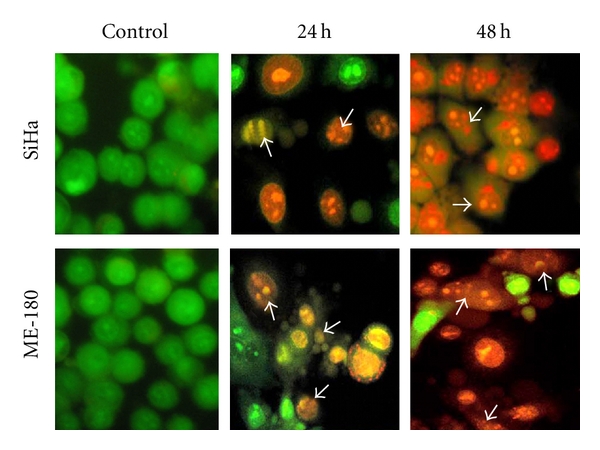
Control and cRGM-treated (24 h, 48 h) SiHa and ME-180 cells stained with acridine orange and ethedium bromide. Arrows point to cells with apoptotic morphology.

**Figure 4:**
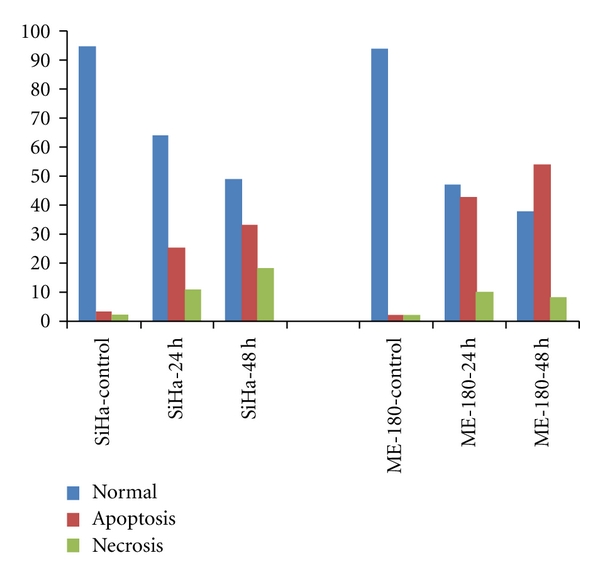
Percentage of normal, apoptotic and necrotic cells at 24 and 48 h in control and cRGM-treated cells.

The results obtained with AO & EB double staining of control and treated cells are presented in the Figure 3. The control cells fluoresced uniformly green and had normal features. Most of the cells treated with cRGM fluoresced red and indicated apoptotic features such as cell shrinkage, chromatin condensation, nuclear fragmentation and apoptotic body formation. A few cells indicated necrotic features such as cell swelling and lysis. Though both SiHa and ME-180 cells responded with higher incidence of apoptosis than necrosis, the incidence of necrosis was higher in SiHa than ME-180 cells (Figure 4). 

### 3.4. DNA Damage as Revealed in Single Cell Gel Electrophoresis

**Figure 5:**
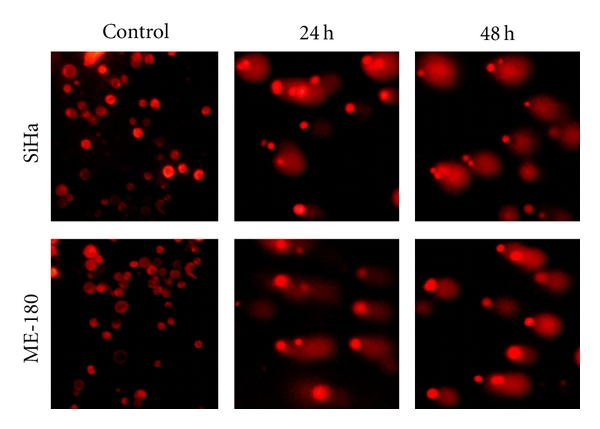
DNA damage in cRGM-treated SiHa, and ME-180 cervical cancer cells as revealed in the comet assay. Comet images of DNA double strand breaks at 24 and 48 h treatment of cRGM.

**Figure 6:**
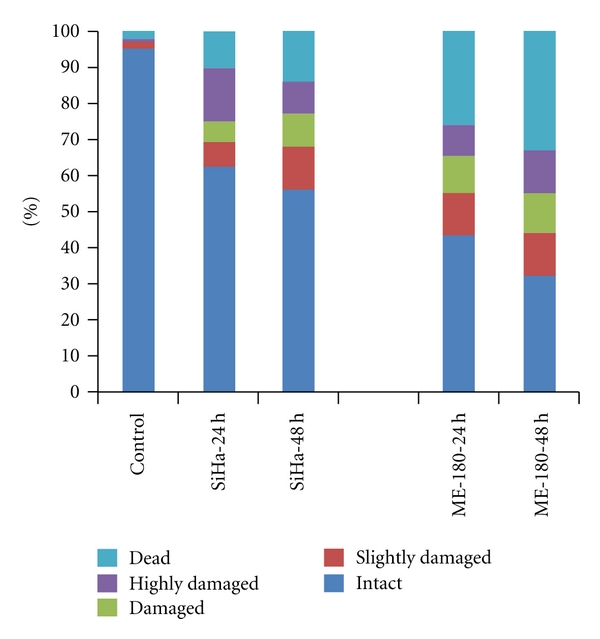
DNA damage in SiHa and ME-180 cervical cells populations as defined according to the DNA in the tail. The multiple parts of each column (from the bottom to the top): intact (0–20%), slightly damaged (20–40%), damaged (40–60%), highly damaged (60–80%), and dead (80–100%).

In order to find if the treatment brings about DNA damage, which is an early event in apoptosis, single cell gel electrophoresis (Comet assay) was conducted. After staining with ethidium bromide and observation under fluorescent microscope (Figure 5), the cells were scored as dead, highly damaged, damaged, slightly damaged and intact, and histograms were prepared using the Comet Analysis Software (CASP) (Figure 6). The chromatin content in the nuclear head, the length of the comet tail, and other comet parameters were recorded for 100 individual cells, and the concurrent comparative data were generated. Though the treatment caused DNA damage to both the cell types, the response was higher in ME-180 cell than SiHa cell. 

### 3.5. Effect of cRGM on Mitochondrial Transmembrane Potential

**Figure 7:**
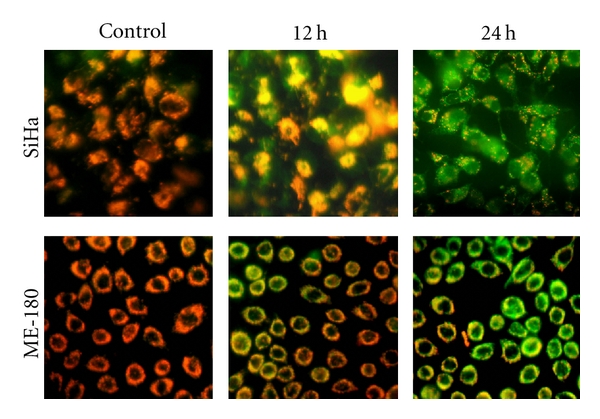
Photomicrographs of SiHa and ME-180 cervical cancer cells. JC-1 dye accumulates in the mitochondria of healthy cells as aggregates (red-orange fluorescing); in cells treated with the cRGM for 12 and 24 h, due to collapse of mitochondrial potential, the JC-1 dye remained in the cytoplasm in its monomeric form, which fluoresced green.

The mitochondrial permeability transition is an important step in the induction of cellular apoptosis. The mitochondrial membrane potential was detected using the unique fluorescent cationic dye, JC-1. The cRGM-treated cells showed progressive loss of red JC-aggregate fluorescence, and appearance of green monomer fluorescence in the cytoplasm at 12 h, and complete loss of red fluorescence presence of only green fluorescence at 24 h (Figure 7). 

### 3.6. Annexin V-Cy3 Assay

**Figure 8:**
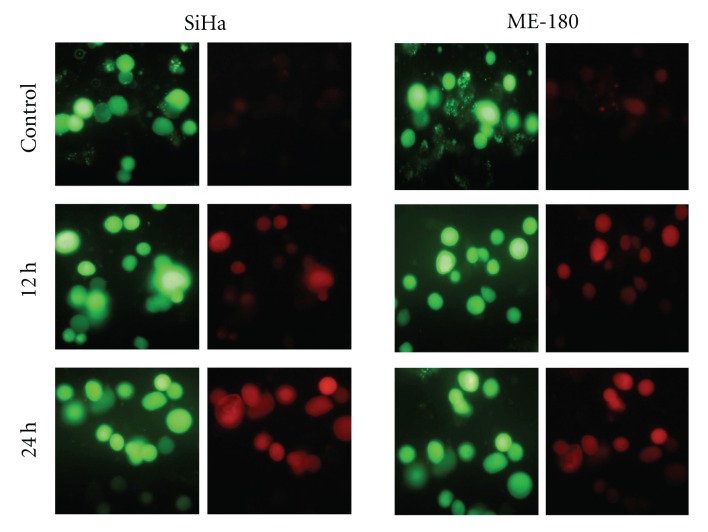
SiHa and ME-180 cervical cancer cells stained with annexin V-Cy3. Cells were treated with the cRGM for 12 and 24 h.

**Figure 9:**
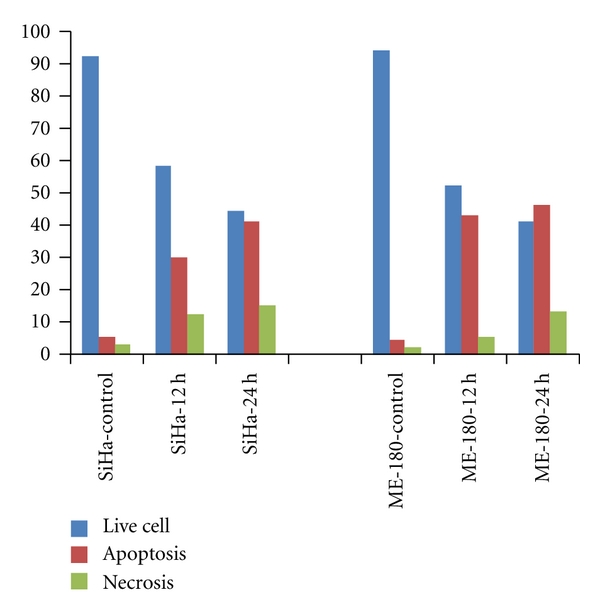
Percentage of SiHa and ME-180 cells in apoptosis and necrosis, control and cRGM treated.

A well-established feature of an early event in apoptosis is externalization of phosphatidyl serine (PS) from inner to outer leaflet of plasma membrane. The results obtained with Annexin V binding assay of control and treated cells are represented in Figure 8. Treatment of SiHa cells with cRGM caused 25 and 33% of cells to succumb to apoptosis during 12 and 24 h treatment, respectively. In the case of ME-180 cell, the corresponding values were higher, 43 and 54%, respectively (Figure 9). In both cell types, a small percentage indicated reflections of necrosis, and the incidence was higher with SiHa than ME 180 cells.

## 4. Discussion

 Since ancient times, plant-based formulations have been practiced as remedies against diverse ailments [15]. Over the past two decades, interest in traditional medicines has increased considerably in many parts of the world [16]. The Indian systems of medicine in general, and Ayurveda and Siddha in particular, which originated several centuries ago, are holistic approaches to healthcare, and RGM is one of the few commonly prescribed medicines for cancer in the Siddha system. The aim of this study was to find if the chloroform extract of RGM, which is not only free from the toxic heavy metal ingredients (which are removed during the extraction process) but amenable for *in vitro* testing, and one which has been already shown to be cytotoxic to PC3, A-549, and H-460 cancer cells, would be cytotoxic to HPV-positive cervical cancer cells, and if so to infer the possible mechanism of action. 

 The outcome of cytotoxicity assay in this study clearly shows that cRGM is cytotoxic to both the HPV-positive cervical cancer cells and produced the effect in very low doses compared to the other extracts, as has been the case with the prostate [7] and lung [9] cancer cells. An earlier study made a preliminary HPLC analysis of cRGM and found about 40–50 compounds in it [7]. Such heterogeneity would provide for the possibility of synergistic and/or additive interactions between the compounds, the sources of which are from the different herbals. Synergism, particularly, is important because it allows lower and safer doses of each compound. Most direct-acting natural compounds, if used alone, would require excessive and unsafe doses to inhibit cancer [17]. The data obtained in this study strongly suggest that, when used in combination, natural compounds can potentially produce synergistic effects *in vitro*. Natural compounds can be divided into three groups: those that inhibit cancer cell proliferation directly, those that act by indirect means to inhibit cancer progression, and those that stimulate the immune system [17]. There is evidence in the scientific literature that the herbals in RGM possess properties such as anticancer, antioxidant, detoxification, and immune modulation. Specifically, the following herbals possess one or more of these property/properties: *Acorus calamus* [18, 19]; *Alpinia galangal* [20]; *Azima tetracantha* [21]; *Celastrus paniculatus* [22]; *Cinnamomum zeylanicum* [23, 24]; *Clerodendron serratum* [25]; *Cocos nucifera* [26]; C*uminum cyminum* [27]; *Curcuma longa* [3, 28]; *Elettaria cardamomum* [24, 29]; *Embelia ribes * [30, 31]; *Foeniculum vulgare* [32, 33]; *Hygrophila auriculata* [34]; *Myristica fragrans *[35]; *Nigella sativa *[3, 36, 37];* Piper longum* [38]; *Piper nigrum *[39]; *Plumbago zeylanica *[40, 41];* Psoralea corylifolia *[42]; *Quercus infectoria *[43]; *Saussurea lappa* [44]; *Semecarpus anacardium *[3, 45, 46]; *Sesamum indicum* [47]; *Smilax china *[48, 49]; *Strychnos nux*-*vomica *[50, 51]; *Strychnos potatorum* [52]; *Terminalia chebula* [53, 54]; *Trachyspermum ammi* [55]; *Vernonia anthelmintica* [56]; *Vitis vinifera *[57]; *Withania somnifera* [58]; *Zingiber officinale* [59–61]. Thus, cRGM presents a strong case for synergism as well as additivism of the multiplicity of compounds from the 38 herbals, most of which have been scientifically proven as associated with one or more aspects of interference with cancer. 

 The idea that an integrated approach is needed to manage cancer using the growing body of knowledge gained through scientific developments [3] is adequately taken care of in our approach of herbal medicine to cancer. Our finding is to be viewed in the background that synergistic interactions occur within a total extract of a single herb, as well as between different herbs in a formulation [62]. In fact, the formulations of traditional medicines used in China, India, and Japan have been constructed to expect desirable treatment of diseases. The principles are based on the interaction of several crude drugs or several ingredients even in a single crude drug. Therefore, the apparent combined effects are equivalent to the sum of effects of those components which underwent addition, potentiation, subtraction, and modulation [63]. 

 Phytotherapy, the therapeutic efficacy of which is based on the combined action of a mixture of constituents, offers new treatment opportunities. Because of their biological defense function, plant secondary metabolites act by targeting and disrupting the cell membrane, by binding and inhibiting specific proteins or they adhere to or intercalate into RNA or DNA [64]. Cancer, by etiology, is multifactorial in origin and, hence, it is only logical that multiple drugs are used at a time. 

 The focus of the present study has been to find if cRGM would inhibit the proliferation of and induce apoptosis in HPV-positive cervical cancer cells, because these are the two major goals in cancer treatment [9]. This study provides evidence in support of the mode of cell death is essentially apoptosis is revealed in features such as chromatin condensation, nuclear fragmentation, and formation of apoptotic bodies. DNA fragmentation is one of the major events in apoptosis. The result of comet assay strongly suggests that cRGM brings about strand breaks in DNA of the cervical cancer cells. The mitochondrial permeability transition is an important step in the induction of cellular apoptosis, and the results clearly suggest that cRGM leads to collapse of the mitochondrial transmembrane potential in cervical cancer cells. This collapse is thought to occur through formation of pores in the mitochondria by dimerized Bax or activated Bid, Bak, or Bad proteins. Activation of these proapoptotic proteins is accompanied by release of cytochrome c into the cytoplasm, which would promote the activation of caspases which are directly responsible for apoptosis [65]. The phosphatidyl serine (PS) expression on the outer leaflet of plasma membrane was detected with annexinV-Cy3 binding, confirming the early stage of apoptosis. Based on the mitochondrial transmembrane potential depolarization assessment, it is reasonable to conclude that cRGM induces apoptosis through the mitochondria-mediated pathway. Study in the future to examine the proteins regulating mitochondria-mediated apoptosis pathway, such as cytochrome c, Apaf-1, adenosine triphosphate, caspase-9, caspase-8, caspase-3, and IAP, will be highly relevant 

 Cervical cancer takes the lives of more than 250,000 women each year globally [66], and most of the cervical cancers are associated with human papilloma virus (HPV) infection [67]. The HPV 16 and 18 oncoproteins E6 and E7 cause immortalization of the infected cells by interacting with and degrading p53 and the cell cycle regulator proteins such as Rb, p21, and p27 [68, 69]. Since cRGM is highly efficient in inducing death of HPV-positive cervical cancer cells, it could be speculated that the extract might restore p53 and the cell cycle regulatory proteins to functional status by causing degradation of viral onco-proteins, which is worthy of investigation. 

 The major limitations of the Indian traditional medicines in presence of one or more toxic heavy metals in the preparations, some intentionally included in view of proprietary prescription in the original formulation (as in RGM), and/or presence of toxic heavy metals to unknown levels in the herbals that are present in the drug. As far as the first is concerned, it is an established fact that the original prescription requires thorough processing of the metal that detoxifies the metal and makes it into a therapeutic substance [70]. In the recent times, there is a concept that the treatments, to which the metals are subjected to make them into nanoparticles [70]. There is evidence that it is potentially toxic when, macro- or microparticles, heavy metal could be nontoxic and therapeutic when made into nanoparticles [71]. It is unfortunate that some quacks try to economize on the preparation and so do not adopt the prescribed procedures [72]. The presence of heavy metals in the herbal ingredients could be overcome through stringent quality control measures [72]. Even assuming that the heavy metals, present in whatever form, are not acceptable, the present study and a few earlier studies [7, 9] show that, in spite of the limitation of deviating from the holism of the proprietary drug formulation which Regulatory Authorities of Indian systems of medicine may object to, even after extracting out the heavy metals RGM in the chloroform extract is potent enough to deal with cancers, especially prostate, lung, and cervical.

## 5. Conclusion

 The original RGM formulation, if exonerated of heavy metal toxicity, or cRGM, which is free from heavy metals, would be a potential evidence-based complementary and alternative medicine for HPV-positive cervical cancers.

## Figures and Tables

**Table 1:** Inhibitory concentration (IC_50_) of different fractions of RGM in cervical cancer cell lines.

	IC_50_ (*μ*g/mL)			
RGM extract	SiHa cells		ME-180 cells	
	24 h	48 h	24 h	48 h
Hexane	414.5 ± 12.3	319.6 ± 14.4	324.6 ± 19.4	282.4 ± 14.2
Chloroform	55.5 ± 7.5	21.6 ± 5.3	40.3 ± 4.1	15.4 ± 3.2
Ethyl acetate	441.6 ± 22.3	324.7 ± 16.2	373.7 ± 21.4	347.6 ± 15.2
Butanol	460.5 ± 18.3	445.6 ± 14.4	448.4 ± 23.2	391.7 ± 13.4
